# An Overview of Current Solutions for Privacy in the Internet of Things

**DOI:** 10.3389/frai.2022.812732

**Published:** 2022-03-03

**Authors:** Guang Yang

**Affiliations:** Department of Computer Science, Electrical Engineering and Mathematical Sciences, Western Norway University of Applied Sciences, Bergen, Norway

**Keywords:** IoT, privacy, SDN, edge computing, differential privacy, PPDM (privacy preserving data mining), blockchain, holochain

## Abstract

As the Internet of Things (IoT) applications have been introduced into daily life, privacy issues have become significant concerns to users, network service providers, device producers, and related roles. This study provides a high-level introduction of current privacy-preserving solutions in IoT systems within the three phases of data collection, transmission, and storage. In these three phases, the following aspects were examined: (1). security protocols at the physical and data link layers; (2). network solutions; and (3). data storage and sharing approaches. Real-world implementations often involve more than one phase, and numerous technologies are combined to ensure privacy. Thus, an understanding of all phases and their technologies can be helpful for IoT research, design, development, and operation.

## 1. Privacy in IoT

Privacy is a topic with a long history, appeared as early as in ancient Greek philosophical discussions. With the development of modern technologies, discussions of privacy have been across domains such as philosophical, political, sociological, and anthropological, and its scope has also been very much evolved (DeCew, 2018). Many countries specify the right to privacy in constitutions and have established laws to regulate personal information dissemination. Internationally, regulations, industrial conventions, and privacy agreements, such as the European Union's *Data Protection Directive (1995)* and *Data Protection Regulation (2012)*, have been proposed. However, taking subtle differences in personalities and cultural backgrounds into account, privacy is still a delicate topic beneath attempts to generalize. Therefore, under the circumstances of the ever-changing world of technology, we often find ourselves uncertain about our privacy.

Privacy is categorized as physical privacy and information privacy. Information privacy is related to the security of personal information and partially overlaps with data security, which is data protection against unauthorized access during transmission across a network and in storage. On top of the data security, privacy is related to the *social context* of the data (Parent, 1983), as data may contain personal information of an individual. These privacy concerns have increased with the introduction of the Internet of Things (IoT). Personal information leaks can be direct or indirect. Direct leaking of personal information, such as sensitive data, location, and identity, can lead to privacy threats in terms of tracking, localizing, and personalization (Porambage et al., 2016). In indirect data violations, content analysis is used for various data mining methods. Misconduct from IoT system owners (or service owners) can lead to severe direct and indirect privacy leaks.

By benefiting from the convergence of multiple technologies, such as cloud computing, artificial intelligence, fifth-generation (5G) networks, and software-defined networks, the IoT offers various applications covering many domains, including home care, healthcare, logistics, transport, and automated vehicular systems. It opens for a diversity of combinations and possibilities to leverage the connectivity to develop cohesive and optimally functional IoT applications and networks, leading to a considerable volume of personal data being generated, gathered, shared through networks, and subsequently analyzed. According to an estimate from the International Data Corporation, by 2025, 41.6 billion IoT-enabled devices are expected to generate 79.4 zettabytes of data.

This study examines current security and privacy measures in IoT systems from the perspective of data collection, transmission, storage, and sharing. The current technologies used on IoT devices and over the IoT networks are security measures with confidentiality, integrity, availability, authenticity, and accountability. We first investigate the security protocols used at the physical and data link layers in Section 2 and detail possible network solutions in Section 3. In Section 4, we investigate various privacy models and privacy-preserving data mining, and discuss privacy concerns in implementing data-chain structures for IoT applications. In Section 5, we compare the current publications on IoT privacy-related topics and conclude further research directions.

## 2. Architectures and Protocol Stacks

An IoT architecture typically has three layers, namely perception, network, and application layers from the bottom to top. These layers provide data sensing, data transmission, and data processing for heterogeneous IoT devices/applications. The sensors on the devices sense and collect data, such as physical parameters, from the environment; the network layer connects devices to the network to transmit and process the sensor data, and delivers application-specific services to end-users (Zeadally et al., 2019).

**Figure 1:**
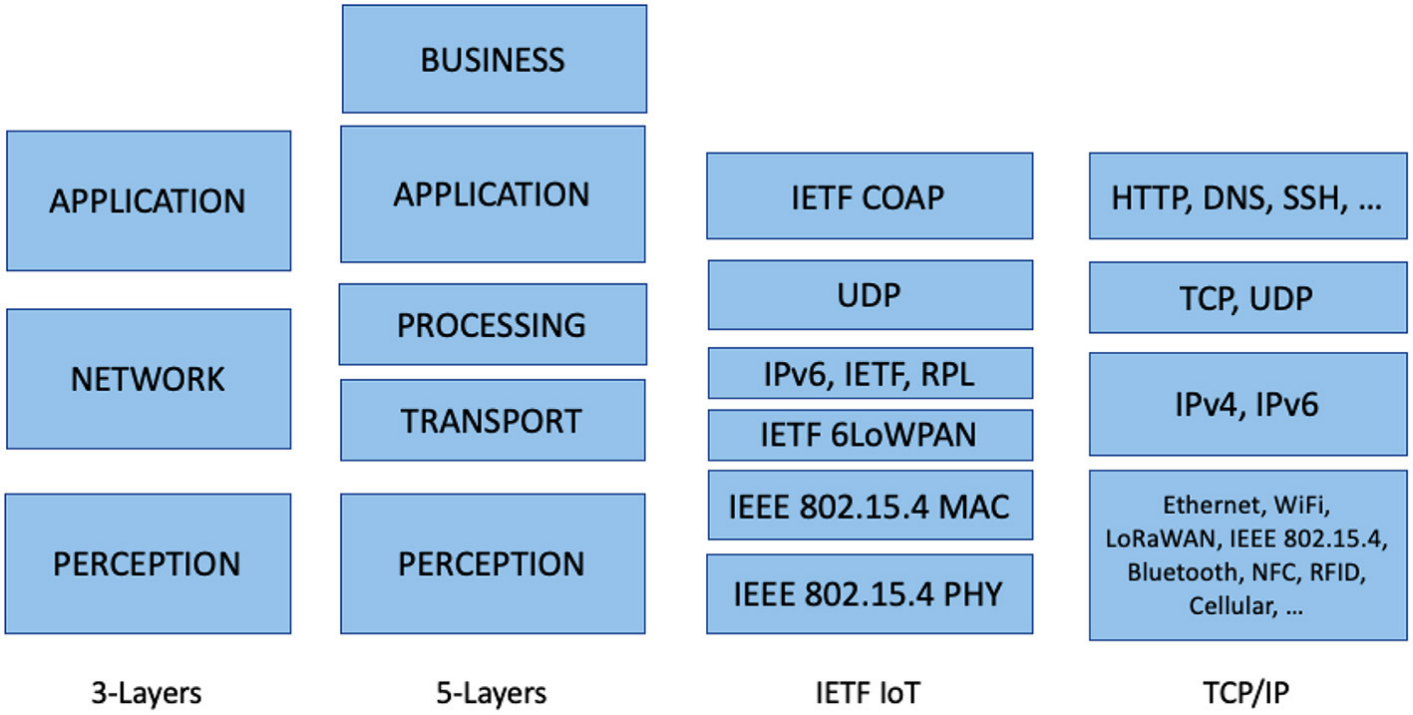
IoT architectures and protocol stacks.

Alternatively, a five-layer architecture was also proposed and referred to. In this architecture, the network layer is categorized into transport and processing layers. In the transport layer, the data are transferred through different networks, and the processing layer stores and processes the data from the transport layer. Databases, cloud computing, and big data processing modules are implemented in the processing layer (Sethi and Sarangi, 2017). In the business layer, data-driven solutions are determined to achieve business goals. This layer is added at the top of the application layer, forming a five-layer architecture (see Figure 1).

Other IoT architectures are similar to the previous two architectures and follow the structure of the standard (OSI or TCP/IP) protocol stacks from the physical layer to the application layer. However, because most IoT devices feature a low capacity of energy and memory, and low-end microcontrollers, some of the existing Internet protocols may not be feasible for IoT implementation. Examples include HTTP and TCP, which are designed to support reliability, and both protocols introduce considerable overhead and non-optimized communication patterns.

Therefore since 2003, numerous Internet Engineering Task Force (IETF) working groups have established a lightweight communication protocol stack for the constrained IoT systems. Protocols in the stack include IPv6 over Low-Power Wireless Personal Area Network (6LoWPAN: RFC 6282), IPv6 Routing Protocol for Low power and Lossy Networks (RPL: RFC 6550), and Constrained Application Protocol (CoAP: RFC 7252), as displayed in Figure 1.

In the IETF IoT stack, the IEEE 802.15.4 standard is used at the physical and MAC layers, and it defines how the physical and media access control layers should operate under low-bandwidth, low-cost, low-speed, and low-energy conditions. 6LoWPAN is a lightweight protocol designed by the IETF to allow IPv6 packets to be transferred over IEEE 802.15.4 networks. RPL is a routing protocol that manages information exchanged among nodes within a local network by using a *destination-oriented directed acyclic graph* topology, which is set up based on a *rank* metric that indicates the distance of each node to its reference root (Palattella et al., 2013). The UDP is used for transport instead of TCP to reduce energy requirements. The IETF constrained RESTful environments working group has defined CoAP, which can be easily translated to HTTP for integration with the web while satisfying specialized requirements, such as multicast support, low overhead, and simplicity, for constrained environments.

At the beginning of establishing this protocol stack, security and privacy are not prioritized. However, some concerns were presented: the RPL ranking algorithm can exclude spoofing nodes from becoming parent nodes, IPsec can be implemented over IPv6, and the CoAP specification has defined four different security modes: NoSec, PreSharedKey, RawPublicKey, and Certificate (Lin and Bergmann, 2016). Subsequently, the IETF working groups in the security domain released solutions in the context of a constrained environment, such as: (a). the DTLS in constrained environment (DICE) (RFC 7925) provides guidelines for using TLS and DTLS in the IoT system; (b). the access in constrained environments (RFC 7744) defines authentication and authorization mechanisms for the entire life cycle of constrained devices; and (c). CBOR object signing and encryption (COSE) (RFC 8125) defines security mechanisms for the CBOR data format, including signatures, message authentication code, and representation of cryptographic keys using CBOR (Morabito and Jimenez, 2020).

In addition to IEEE 802.15.4, wireless air interfaces involved in the IoT include Wi-Fi (IEEE 802.11), LoRaWAN (long-range wide-area network), Bluetooth Low Energy (BLE), near-field communication (NFC), radio-frequency identification (RFID), and cellular/mobile connections (3GPP LTE. NR). These wireless protocols provide ubiquitous wireless connections that can ensure considerable diversity of IoT applications. However, in wireless networks, broadcast renders transmission vulnerable to both passive and active security attacks, and the features of portable and mobile devices pose additional risks.

Security countermeasures at the lower layers of the IoT are mainly categorized into *computational security* and *information-theoretic security* (Zou et al., 2016). Computational security is the primary approach for protecting communication systems where cryptographic algorithms are implemented. Cryptographic schemes are of two types, namely symmetric and asymmetric (public-key cryptography [PKC]). In symmetric encryption algorithms, substitution and transformation operations are performed on the bits based on the key. Asymmetric encryption algorithms are based on mathematical functions, such as discrete logarithms, and the security is dependent on the computational hardness of solving mathematical problems. Typically, PKC is used for key distribution in a crypto system, and session keys are shared for symmetric encryption/decryption.

This solution may not be the most suitable solution for IoT devices with low-cost, low-energy, and lightweight computing requirements. Furthermore, asymmetric schemes are vulnerable to quantum attacks. When sufficiently capable quantum computers are available, PKC schemes can be deciphered unless the key sizes increase to impractical lengths (Zhang et al., 2017). The physical layer security (PLS) relies on information-theoretic proofs of perfect secrecy, was firstly introduced by Shannon (1949), has been considered as an alternative security solution for IoT devices. The information theory is typically implemented in channel coding for ensuring the reliability of digital communication. Channel coding enables the receiver to detect and correct errors introduced during transmission because of noise, interference, and fading. Examples include the *turbo codes* used in 4G and 5G NR utilizing the *polar codes* for the control channels and *LDPC* for the data channels. In theory, a PLS system cannot be compromised, irrespective of the adversarial computational, and its implementation is straightforward.

### 2.1. Key Generation and Distribution

#### 2.1.1. Zigbee

In a secured Zigbee network, security is centralized by the network coordinator (*trust center*), which can authenticate devices that wish to join the network. The *trust center* sends a network key to authenticated devices, and all the nodes from the same network use this key to encrypt/decrypt the general protocol maintenance data and some user data. The *trust center* also provides a link key for any two nodes that wish to communicate using encryption/decryption functions from the application layer. For the devices that run the same ZigBee application, in each Zigbee device, a certificate-based key establishment is used to drive a unique public key (and other security elements) as its identity. In addition to the centralized security network, ZigBee 3.0 allows decentralized security management through a distributed security network.

#### 2.1.2. BLE

BLE devices remain in the sleep mode, except when participating in data exchange (BLE, 2017). Two BLE devices must first authenticate their identities. During pairing, the two devices distribute long-term keys for encryption. When authenticated, the link between the two devices is encrypted, and the keys are distributed. If the keys are saved for future reconnection, the devices are said to be *bounded*. BLE specifies that a connection can be operated in a specific security mode with several security levels.

#### 2.1.3. IEEE 802.11

The IEEE 802.11 wireless working group designed the wired equivalent privacy (WEP) algorithm to ensure data confidentiality in the original 802.11 standards (1997). In 2003, the Wi-Fi Alliance promulgated Wi-Fi Protected Access (WPA) as an intermediate solution to WEP insecurities. In 2004, IEEE 802.11i was ratified as an amendment to the original IEEE 802.11 and implemented as Wi-Fi Protected Access II (WPA2), also called robust security networks (RSNs). The IEEE 802.11i RSN security specification defines the following services: authentication, access control, and privacy with message integrity, by five phases of operations, including the generation and distribution of two types of keys: pairwise and group keys (Stallings, 2017). In 2018, the WPA3 was released with improved security features: a more secure handshake procedure for establishing connections, providing protection for open hotspots, and increasing the key size.

### 2.2. Data Encryption

In both Zigbee and BLE, 128-bit AES-based encryption is used for network communication. The IEEE 802.11i amendment defines two data confidentiality and integrity protocols, namely the temporal key integrity protocol and counter mode with cipher block chaining message authentication code protocol, in which the cipher block chaining message authentication code is used to provide message integrity and the CTR block cipher mode is used with AES for encryption.

Although AES can be swiftly implemented on microcontrollers, they tend to be large and complex. Moreover, the block size may not always be optimal. For example, an RFID authentication protocol may only ask 64-bit quantities to be encrypted (Beaulieu et al., 2015). Therefore, many studies have been conducted to reshape the AES into a lightweight solution for IoT applications. In Hogan and Piccarreta (2018), NIST-approved lightweight cryptography standards include ISO/IEC 29192-(1-5), cover block cipher, stream cipher, mechanisms using asymmetric techniques, and hash function.

### 2.3. PLS

The objective of PLS is to investigate the physical properties of the communication channel to enhance security through appropriate coding and signal processing. According to Shannon's theory, the achievable secrecy rate is a function of the channel properties and the block length of the encoders. Thus, security is related to the properties of communication channels.

In the physical layer key generation, the randomness of channels (the characteristic features), which involves channel probing, quantization, information reconciliation, and privacy amplification, are considered. The process does not necessitate using a third party, and it is lightweight and requires limited resources. Therefore, physical key generation can be used as an alternative to PKC in many cases, especially in IoT devices. In Shakiba-Herfeh et al. (2020), the feasibility of moving security core functions (node authentication, message integrity, and message confidentiality) down to the physical layer was investigated by using both the communication radio channel and the hardware as unique entropy sources. In Zhang et al. (2017), a hybrid approach that is constructed by physical layer key generation and physical layer encryption, which performs encryption operations at modulation stages of the physical layer, was used to protect the IoT system.

Many PLS transmission schemes are not practical because their security benefits theoretically rely on an idealized simplifying assumption, such as the availability of perfect channel state information knowledge and the development of coding (Zou et al., 2016).

## 3. Network Solutions

Most IoT networks are cloud-centric solutions in which cloud servers are the primary storage of user data and the center of security services. With the exponential increase in the data volume and the number of connected IoT devices, network issues, such as high latency, bandwidth bottlenecks, and scalability energy within this paradigm, have emerged. Thus, fog computing and edge computing, in which computation and data storage are close to end-devices, were subsequently introduced into IoT networks. Security services, such as identification and authentication, were consequently moved from the cloud to the fog and edge layers.

The network's privacy threats can be traffic analysis, eavesdropping, and attacks like Man-in-the-Middle, DoS, and DDoS are susceptible to IoT networks. Currently, authentication of devices and key management mechanisms to prevent communication channels from being compromised are vital security measures for IoT networks. Because edge servers consist of heterogeneous and distributed devices (or infrastructures) with limited computing and storage capabilities, the security services should be lightweight designed. Notably, the identification and authentication of users and the confidentiality of user data are typically handled differently by applications from the application layer.

**Figure 2:**
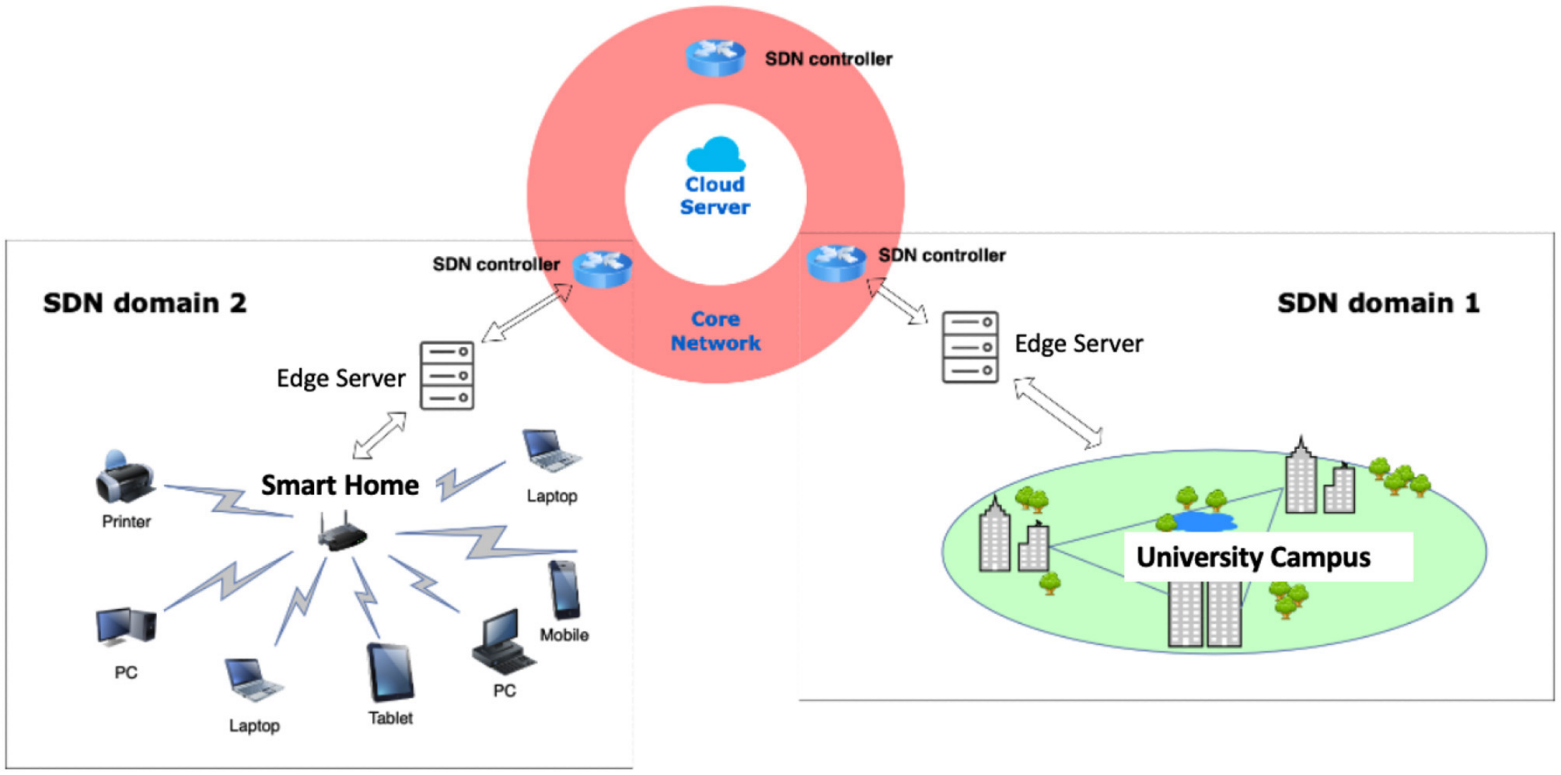
An example of SDN solution of IoT networks.

We propose a general solution for IoT networks by introducing a software-defined network (SDN) that enables a dynamic, programmable network configuration. By separating the control and data planes, SDN controllers have a global view of the network that helps traffic engineering identify malicious traffic patterns by developing and implementing different network functions as SDN applications. Thus, the networks can be more resource-efficient and resilient to attacks. We describe the IoT networks by local networks, edge servers, a core network, and cloud servers, as shown in Figure 2. A local area network (LAN) connects heterogeneous IoT devices based on their IP addresses at the local network layer. Devices are associated with a close edge server connected to the core network. The nodes at the core network layer are primary SDN controllers as the coordinators of their SDN domain, defined as local networks operated by the same SDN controller at the core network layer. SDN domains ensure effective management to enforce security and privacy policies, such as access control, authentication, and domain. In addition, data-chain structures, such as blockchain, can be applied within a domain or among domains as required. An example of the SDN is displayed in Figure 2, which illustrates SDN domain 1 is a University campus network that can be several buildings at different geographical locations, and all LANs are connected to the same SDN controller at the core network layer. SDN domain 2 is a smart home solution connected to another SDN controller at the core network layer.

## 4. Data Storage and Sharing

Typically, data collected or generated from local IoT devices are transmitted and stored in the cloud (some data/metadata may be kept at the edge/fog layer). Depending on the structure and usage of data, storage can be realized through relational databases, NoSQL databases, and data lakes. Cloud services offer personal data storage to individual users and data sharing possibilities for business collaboration. Under these circumstances, private data are vulnerable to malicious attacks and misconduct from service providers. For the best use of the data, models that can abstract global statistical information or apply certain data mining algorithms for information sharing without revealing user privacy become exigencies of the IoT. For other security concerns about recording the events/transactions occur in the network, a novel data structure, chain structure, has drawn considerable attention from both industrial and academic research.

### 4.1. Differential Privacy

Cynthia Dwork defined the differential privacy in Dwork (2006), it is dealing with present statistical properties from a database while protecting individual's privacy that is contained in the database. In differential privacy, by introducing noise, a single arbitrary substitution has a minimal effect on the global statistical output; thus, arbitrary queries cannot track any individual and provide privacy.

Wang et al. (2019) demonstrates an edge-based differential data collecting scheme for sensor-cloud systems. A set of raw data is categorized into metadata and residuals after being collected from a local device and transmitted to an edge server. The edge server encrypts the two parts of the data (using the AES-Reed-Solomon code). The encoded residual data are uploaded and stored in the cloud. Users can decide whether the encoded metadata is stored at the edge server, the local device, or partially on each server. This approach prevents the data from being compromised or the raw data being revealed in the cloud. Two algorithms were adopted to select appropriate metadata and calculate the corresponding residuals: (1). root mean squared error is used to determine a small degree of deviation between the metadata and residuals; and (2) based on *K*-means clustering algorithms, a data set with multidimensional attributes is divided into subsets according to their similarity, and cluster centers are determined as the metadata, and the distances from the cluster center to the cluster are minimized as residuals. In Yin et al. (2018), the authors introduced location privacy protection for big data in industrial IoT by using differential privacy. This study constructed a tree structure using multilevel location data, determined accessing frequent patterns of locations, and added Laplace distribution base noise to the frequent patterns. The differential privacy protection model was applied to protect location privacy and maintain data availability.

### 4.2. Privacy-Preserving Data Mining

Data mining is the process of extracting data patterns. Typical data mining examples that breach privacy include location pattern mining, identification mining, and sensitive text context mining. To address the privacy concerns in data mining, privacy-preserving data mining (PPDM) protects sensitive information from unsolicited or unsanctioned disclosure and preserves the use of the data (Xu et al., 2014). PPDM is used to modify the original data so that data mining algorithms can efficiently perform without compromising the privacy of the individual contained in the data. The modifications include perturbation, blocking, aggregation, and swapping. Moreover, numerous privacy preservation methods, such as *K*-anonymity, classification, clustering, association rule, distributed privacy preservation, L-diversity, randomization, taxonomy tree, condensation, and cryptography, have been devised for data mining (Sachan et al., 2013). Distributed PPDM (DPPDM) methods can be implemented for distributed data storage and sharing environments. Existing DPPDM techniques are categorized into three groups, namely secure multiparty computation, perturbation, and restricted query (Aldeen et al., 2015). Other techniques such as game theory and rational multiparty computation have been proposed for addressing privacy concerns in IoT applications (Du J. et al., 2019; Du M. et al., 2019; Butpheng et al., 2020).

### 4.3. Chain Structures

#### 4.3.1. Blockchain

The blockchain is essentially a distributed ledger that multiple participants jointly maintain. The ledger is a chain of blocks that are time-stamped and linked by cryptographic hash functions, as each block holds the previous block's hash value. The cryptographic linkage among blocks in the chain results in the properties of the blockchain being *append-only* and *immutable*. The chain is duplicated across the network and distributed by a group of nodes, such as *mining* nodes, which create and append new blocks to the chain according to a predefined consensus mechanism. These properties ensure the integrity of the data and resistance to the tempering of transactions by design. Because all transactions are stored and shared by all nodes in a decentralized fashion, blockchains provide new possibilities for the interoperability of the network. Therefore, blockchain-based architectures have been proposed for many IoT applications to provide superior security and improve the interoperability of systems. The most prevalent implementation is in the E-healthcare system, in which the patients' health records are chronological and commonly shared by authorized healthcare institutes.

However, privacy concerns remain when implementing blockchain in IoT systems. Blockchain networks ensure the anonymity of users participating in transactions using pseudonyms. For example, for the Bitcoin payment system, when a user joins the network, he/she obtains a Bitcoin account, which is defined by an elliptic curve key pair where the public key is used to generate a Bitcoin address as its public identity, and the corresponding private key held by the user to spend Bitcoin from this account (Herrera-Joancomarti, 2014). Anonymity in the Bitcoin network is based on users' ability to create any number of Bitcoin accounts for their transactions.

A transaction records a certain amount of Bitcoins from a source address to a destination address. Although the addresses are pseudonyms, the amount of Bitcoins is indicated in the plaintext in any transaction. To prevent double-sending attacks, each transaction is broadcasted to the network, and every user must validate the transaction. Finally, all transactions are recorded on the blockchain permanently. It is possible to reveal information such as the usage pattern of a particular account or the usage pattern between two certain accounts. The worse thing is possible to find out a real-world identity associated with the Bitcoin accounts. Since a single user can have many accounts, the attacker's strategy is to identify a cluster of addresses in the blockchain system belonging to the same user. In Reid and Harrian (2011), a transaction network and a user network were constructed from Bitcoin's public transaction history, with external information and techniques such as context discovery and flow analysis on these two networks. The results revealed that many Bitcoin addresses could be associated with each other, with external identifying information, and users' activities can be observed in detail. In Androulaki et al. (2013), the authors simulated the Bitcoin system used as a primary currency for daily transactions of individuals in a university setting. The results revealed that the profiles of almost 40% of the users could be unveiled.

#### 4.3.2. Holochain

As an alternative to the blockchain, implementations of holochain in the IoT systems have been discussed and explored recently. Holochain is an application framework where a peer-to-peer application, namely hApp is developed and run on. In holochain, the use of an agent-centric approach allows the user (agent) of hApp to have autonomy, as the user maintains a private source chain of his/her transactions locally. Peers in the network validate transactions by utilizing a distributed hash table (DHT) that is unique in the network and shared among the peers (Brock et al., 2021). Each agent of a holochain network stores the information of every transaction with the validation rules and application source code locally. Similar to the blockchain, to ensure the integrity of transaction data and prevent tempering, holochain exhibits a chain structure for data blocks and generates a hash value as the signature for each block. Each block contains the hash value of the previous block. However, unlike blockchain, the same chain is duplicated among the P2P network; the holochain is stored locally as a private source chain on each agent's device. An agent of the holochain only maintains transactions that have happened to him/her. Each agent stores transaction data locally in the source chain and publishes the headers of transactions (and other public data) to a random selection of peers for validation. If the transaction is valid, the peers store the copy and share it with their neighbors. The holochain network holds a DHT, which is a key/value table for all valid shard headers, and each node holds a small shard of the DHT^1^. In terms of privacy, the individual agent has authority over data sharing, access, and storage through the hApp. This agent-centric approach provides superior control of personal data compared with data-centric approaches (Wahlstrom et al., 2020).

In Janjua et al. (2020), a fog computing-based architecture was proposed in which log collection from an IoT environment is automated and secured. The architecture consists of three layers. In addition to the log generation and collection layer, the log preservation layer uses holochain to preserve the log in fog nodes to ensure the integrity of logs and provide temper resistance. In their design, the fog nodes receive the logs from edge nodes, create information digests of logs, and store them locally on the chain, whereas the log files are eventually stored in the log archiving layer (the cloud). This design also provides trust admissibility and ownership nonrepudiation of logs. The investigator can verify the logs stored on the cloud by recovering the digests stored on the fog node holochains and DHT from the log preservation layer.

## 5. Discussion and Conclusion

Privacy is a concern in the modern technological world. With the advent of IoT and its widespread, privacy concerns have increased manifold. In general, for any IoT application, data are collected from the perception layer, transmitted across networks, and stored in a data storage. This study reviewed the privacy concerns and current methods used at each phase to ensure IoT privacy. At the physical and data link layers of communication, during the data collection phase, privacy is offered by standardized data security protocols (such as encryption, authentication, and key distribution). In cases where the security protocols are not suitable for the IoT devices or can not satisfy the requirements of the application, then specific encryption, authentication, or key distribution algorithms are proposed. Thus, the critical privacy concerns at this phase remain on indirect privacy leaks, such as finding users' behavior patterns, and protecting the user anonymity. In the data transmission and storing/sharing phases, the individual system design is performed according to specific policies or rules that provide privacy to the system users.

**Figure 3:**
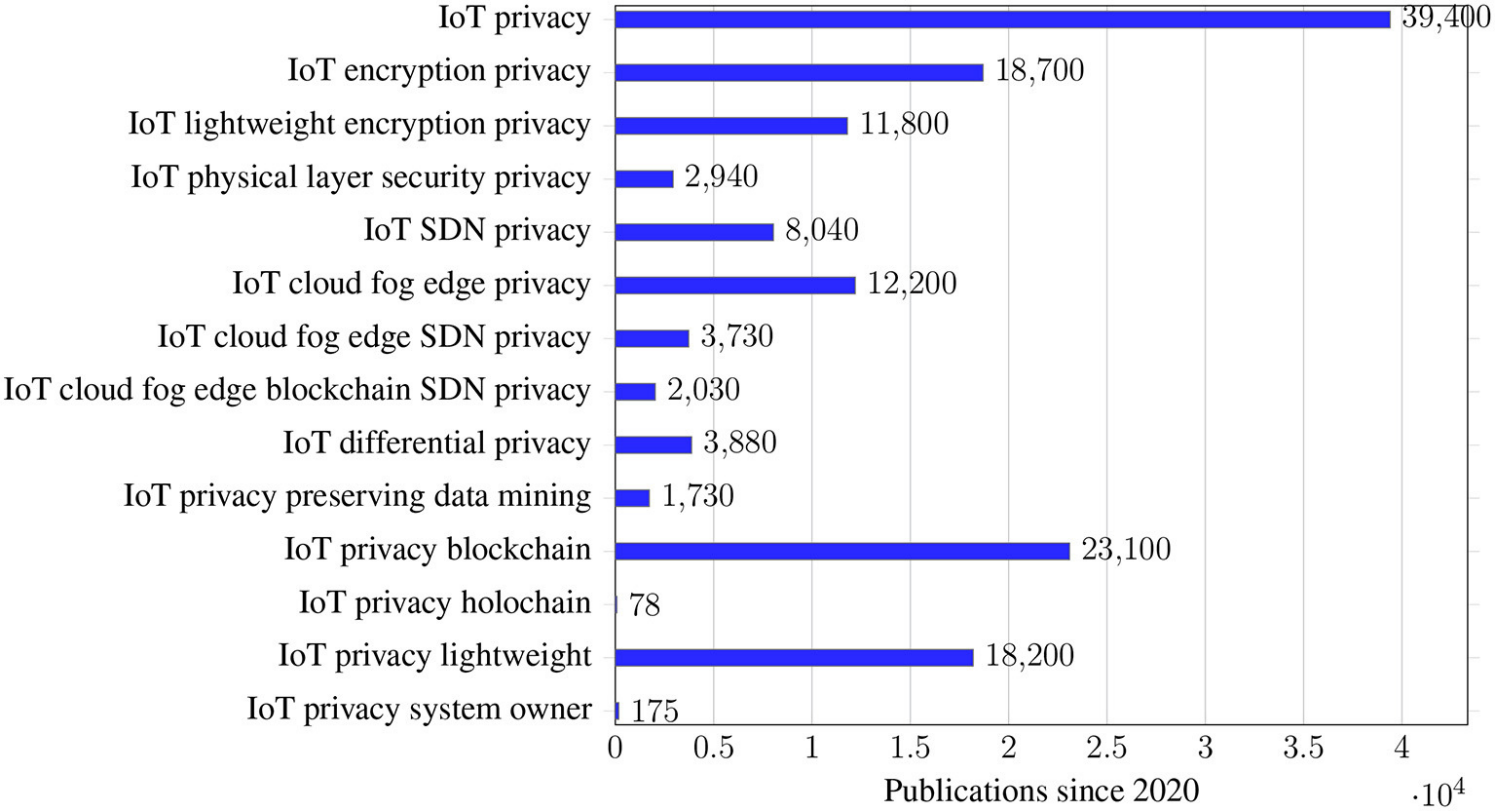
Total publications by research topics from 2020 to 2021 (from *Google Scholar*).

We often see a combination of technologies in actual implementations or proposed research solutions, with privacy concerns for each component. For example, design lightweight encryption/authentication methods on the IoT devices/network, use fog edge structure and cloud storage as the network architecture, and use blockchain for anti-tampering, tacking logs, or improving system inter-operability. In Figure 3, a statistical overview of publications on IoT privacy research is listed from *Google Scholar* from 2020 to the end of 2021^2^. Many IoT security/privacy solutions have been developed, especially in data storage and sharing, and are application-specific. We have seen different IoT applications have various implementations of differential privacy and PPDM. Thus comparing the implementations and summarizing the essentials will be necessary for future work.

Although most solutions emphasize the *lightweight* of implementation, this may quickly result in an inefficient and over-complex IoT ecosystem. If proposing a generalized paradigm is not feasible because of the fast development of the involved technologies, modularization could help in this situation. And, we also notice that the most significant challenge, the leaking of user privacy from IoT system owners (or service owners), has not been addressed sufficiently. Since the network and data storage are developed and belong to system owners, users have very limited data autonomy and often have limited knowledge about their data. When the functionality of an IoT application is more critical to the user, such as some healthcare applications, users can only compromise or risk their privacy since they have no choice. Thus further research in this direction on data storage and sharing is critical, especially the user data autonomy.

## Data Availability Statement

The original contributions presented in the study are included in the article/supplementary material, further inquiries can be directed to the corresponding author/s.

## Author Contributions

The author confirms being the sole contributor of this work and has approved it for publication.

## Conflict of Interest

The author declares that the research was conducted in the absence of any commercial or financial relationships that could be construed as a potential conflict of interest.

## Publisher's Note

All claims expressed in this article are solely those of the authors and do not necessarily represent those of their affiliated organizations, or those of the publisher, the editors and the reviewers. Any product that may be evaluated in this article, or claim that may be made by its manufacturer, is not guaranteed or endorsed by the publisher.

## Footnotes

^1^ https://developer.holochain.org/concepts/

^2^The results are a total number of publications by using *advanced search* of words in *Google Scholar* from 2020 to November of 2021 (on IoT privacy, there were 39,400 publications). Google Scholar does not cover all the publications. The order of words matters results because of the search algorithms, and search by words unnecessary return the publications are all on that explicit topic of the words. The figure displays approximately and comparable results.
